# The young adult hip: extra-articular causes of hip pain and how to pick the winners

**DOI:** 10.1093/jhps/hnv012

**Published:** 2015-02-18

**Authors:** Edward D. R. Bray, Milad Sherafati, Charlotte L. Cutts, Giles H. Stafford

**Affiliations:** Research Department, The Elective Orthopaedic Centre, Epsom KT18 7EG, UK

## Abstract

Hip pain in young adults is not always caused by intra-articular pathology, even in the presence of abnormal examination and imaging findings. Therefore, management of young adult hip pain requires processes that identify patients who are likely to benefit from surgical intervention. An important investigation in the diagnostic pathway is the intra-articular injection; a negative response to this should alert the surgeon to the presence of symptomatic extra-articular causes of hip pain. Our aim was to identify the proportion of patients referred with intra-articular pathology whose primary cause of pain was of extra-articular origin. A total of 143 intra-articular hip injections (local anaesthetic + corticosteroid) were performed over a 2-year period. Mean patient age was 41.95 (95% confidence interval: 39.50–44.41) years with a mean body mass index of 27 (95% confidence interval: 25.77–28.23); 26% of patients (*n* = 37) had no relief of symptoms after intra-articular injection. Of the patients with no relief, 81.1% (*n* = 30) were found to have extra-articular pathology as the cause of their pain and the remainder are under on-going investigation. Intra-articular hip injection is an important investigation in the diagnostic pathway of young adult hip pain, as it can highlight and differentiate those patients with referred pain from extra-articular pathology. This benefit may be further enhanced if injections are performed in theatres using image intensifier, under sedation, as it allows direct penetration into the joint without any local anaesthetic infiltration of surrounding tissue. The latter allows immediate objective assessment of symptom relief, eliminating the need to rely on retrospective patient recall of symptom change.

## INTRODUCTION

The young adult presenting with hip pain often reports relatively non-specific symptoms and on physical examination, can be difficult to elucidate the precise driver of their pain as innervation is broad and ill-defined. Consequently, patients are unable to pinpoint the exact location of their symptoms due to referred pain, and therefore, the aetiology of the pain cannot always be clear [1].

**Fig. 1.**
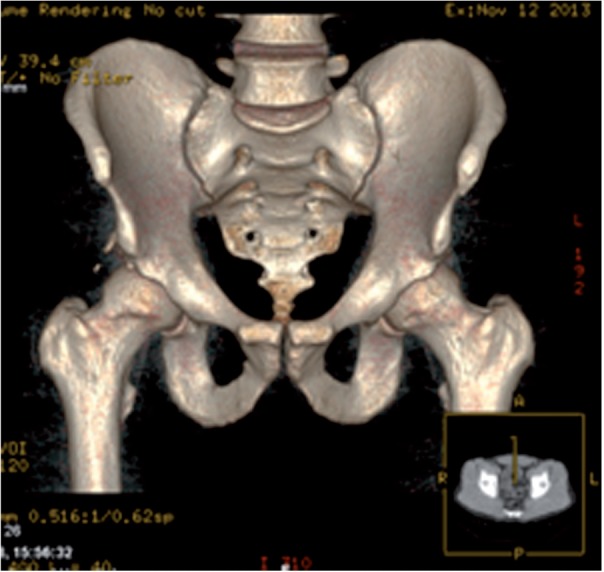
Three-dimensional reconstruction of computed tomography scan.

**Fig. 2.**
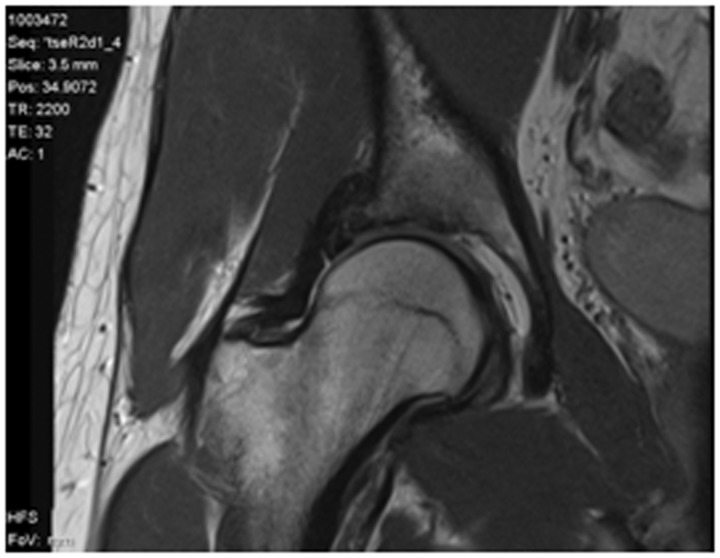
Slice from 3 Tesla MRI scan.

In patients with no significant arthritic change on plain weight bearing radiographs, we typically investigate further with computed tomography and magnetic resonance imaging (MRI) scans (Figs. 1 and 2). As diagnostic imaging has improved, particularly with respect to MRI techniques, the differential diagnoses have broadened. A surgeon may be faced with more than one potential diagnosis and a resultant diagnostic dilemma. These differentials may be intra-articular hip pathology, extra-articular pathology or pathology unrelated to the hip itself. Consequently, management of young adult hip pathology requires processes that identify patients that are likely to benefit from orthopaedic surgical intervention [1].

For over half a century, intra-articular injection of corticosteroid has been used by orthopaedic surgeons and rheumatologists to provide symptomatic relief [2–4]. In addition to providing this temporary relief, the intra-articular injection comprising a combination of local anaesthetic (LA) and corticosteroid is a useful tool in our diagnostic armoury. Symptomatic relief following such an injection indicates a positive result and confirms that the pathology driving a patient’s pain is of intra-articular origin.

It could be said that it is more important to a surgeon if a patient has pathology on diagnostic imaging but gains no relief from the injection. This indicates to the surgeon that the pathology identified on the patients’ imaging is incidental, therefore ensuring that the surgeon does not perform an unnecessary operation by not treating the root cause of their pain. Furthermore, it ensures that other avenues are explored to establish the true cause of a patient’s symptoms.

## AIM

To identify the proportion of patients referred with intra-articular pathology whose primary cause of pain was of extra-articular origin.

## METHODS

Using Bluespier (Bluespier International, Worcestershire) we searched for all intra-articular injections of LA and corticosteroid into the hip performed between April 2012 and April 2014 undertaken by a single orthopaedic surgeon with a specialist interest in young adult hip surgery. Using an audit tool, the following information was recorded on all patients: age; body mass index (BMI); whether they gained pain relief; duration of pain relief; complications; further investigation results and management. Confidence intervals (CIs) of the means were performed using GraphPad (GraphPad, CA) Statistics software.

This work was registered with the audit department at The EOC, Epsom, Surrey, UK.

## RESULTS

Between April 2012 and April 2014, a total of 143 intra-articular hip injections of LA and corticosteroid were performed either under ultrasound guidance (120) or in theatre using an image intensifier (23). The mean age of the group was 41.95 (95% CI: 39.50–44.41) years with a mean BMI of 27 (95% CI: 25.77–28.23). Complete pain relief was seen in 104 (72%) patients, lasting an average of 5.0 weeks (95% CI: 4.15–5.89). Out of our cohort, three different complications were noted: one infected joint (ultrasound guided), two transient femoral nerve palsies (ultrasound guided) and five significant steroid flares.

No relief from the injection was seen in 37 [26% (95% CI: 19.37–33.64)] patients, 31 of those having undergone injection under ultrasound guidance (see Table I for comparison of technique). The pre-operative differential diagnoses of these patients were labral tears. After excluding the hip joint as the primary source of pain, the extra-articular pathology discovered on further investigations included degenerative spinal problems (*n* = 14), greater trochanteric pain syndrome (GTPS) radiating to the groin (*n* = 8), inguinal hernias (*n* = 2), rectus avulsions (*n* = 1), osteitis pubis (*n* = 1), neurological (*n* = 1), urological (*n* = 1) and gynaecological (*n* = 2) diagnoses. Seven patients who did not gain relief from the investigation are still under investigation; these injections were performed under ultrasound guidance and the patients are awaiting repeat injections in theatre as no further pathology has been found.

**Table I.** Comparison of injection techniques used

US guided	Theatre using image intensifier
Performed by consultant radiologist	Performed by consultant orthopaedic surgeon
Supine position	Supine position
Aseptic technique	Alcoholic chlorhexidine skin preparation
±2 ml of lignocaine	No soft tissue LA
80 mg of Kenalog (triamcinolone) + ∼10 ml of bupivacaine (0.5%)	40–80 mg methylprednisolone (depending on body habitus) + 10 ml Bupivacaine (0.5%)
US guidance (in radiology department)	Image intensifier (in theatre)

A useful assessment of the ability to differentiate patients with symptomatic extra-articular pathology is the negative predictive value of the intra-articular hip injection. From our cohort, 37 patients had no relief after their injection, and out of those, 30 were found to have an extra-articular pathology as the primary cause for their pain. Defining those with no other pathology as false negatives, this creates a negative predictive value of 0.81, which means 8 out of 10 patients who find no relief from the hip injection will have an extra-articular cause for their symptoms.

## DISCUSSION

Our study shows that approximately a quarter of our cohort gained no relief from the injection despite having intra-articular pathology on diagnostic imaging. As a result, these patients have avoided an unnecessary operation and the potential hazards associated with this. Furthermore, the ability to exclude the hip joint as a source of their pain ensures that we as diagnosticians look closely at the aetiology of their pain and thus address the pathology causing this pain.

The most common symptomatic extra-articular pathology in patients with no relief from injection was either spinal pathology or GTPS.

The lack of pain localization and complex regional anatomy mean that there are a number of pathologies related to the spine, which can result in groin pain. Herniation of the annulus pulposus, particularly at the level of L4/5, has been shown to result in groin pain with the spinal team investigating this agreeing that the intra-articular injection into the hip is a useful diagnostic tool [5, 6]. A dull anterior thigh pain may be the result of intrinsic hip pain, but spondylolisthesis has also been shown to present in this manner [7]. Furthermore, lumbar facet syndrome also presents in a similar fashion to hip pain with pain around the paralumbar facets and buttock which refers to the greater trochanter, posterior thigh and into the deep groin [8, 9].

GTPS includes a number pathologies relating to the lateral hip and its soft tissues [10]. These vary from greater trochanteric bursitis to tearing or tendonitis of the abductor muscles [11]. Those with trochanteric bursitis may report pain over the greater trochanter radiating down the lateral thigh [12], although patients with true bursal inflammation contribute to a small percentage of GTPS aetiology [13]. Additionally, intra-articular pathology has been found to predispose patients to GTPS [11]. This confounding prevalence can create difficulties in diagnosis when intra-articular pathology is highlighted on imaging, in addition to a component of GTPS [14, 15]. In such cases, the intra-articular injection is useful in establishing the true pain generator and therefore the area that requires definitive treatment.

Iliopsoas-related pain is another common extra-articular cause of groin pain [16]. Ranging from tendonitis to coxa saltans, patients frequently report anterior groin pain associated with extending the hip from a flexed position and may be accompanied by intermittent ‘snapping’ of the hip [12, 16]. With a higher prevalence amongst those who undertake sports, especially runners, as well as young individuals generally [17, 18], this diagnosis is an important differential that could mimic hip pain in this patient group.

Other orthopaedic causes of hip pain include rectus avulsion (complete or partial), which are predominantly seen in athletes. These can present similarly to labral tears, whereby the patient undergoes a traumatic event and experiences anterior thigh pain and a limitation in range of movement caused by pain inhibition. The management of this pathology is clearly different, and it is therefore critical to establish the true cause of pain at an early stage [19–21].

Both inguinal and femoral herniae are causes of groin pain and as such should be considered in the differential diagnosis of those presenting with diffuse groin pain [9]. Furthermore, there are gynaecological aetiologies that can also become symptomatic in the form of hip pain. These include retroperitoneal endometriosis and large ovarian cysts which can result in referred pain from the abdomen or pelvis to the hip [22].

This study aimed to establish whether the intra-articular hip injection is a useful diagnostic tool to aid the management of ‘hip or groin pain’. We believe that the ability to establish whether pain generators are intra- or extra-articular is critical in the appropriate management of these patients. The study’s retrospective nature enabled us to review our current practice, allowing us to establish whether this additional information is affecting our management. Our data have shown that approximately a quarter of our patients who had labral tears did not respond to the injection and as such are not suitable candidates for hip arthroscopy to treat the cause, in most cases femoroacetabular impingement.

Our data show a strong negative predictive value of 0.81 for patients who do not respond to treatment. Although this is a significant number in itself, the true value might be even higher as our definition of false negatives might have underestimated the predictive power of this diagnostic test. The reason for this is that these patients had their injections under ultrasound guidance and are awaiting repeat injections in theatres. If they are found to have relief from the repeat injection, then there may have been inadequate penetration into the joint, which can have acted as a confounding factor in this context.

However, there are risks and side effects relating to the intra-articular injection. Firstly, there is a small but significant risk of intra-articular sepsis, which has been demonstrated in the complications of our cohort. Furthermore, a steroid flare, whereby there is crystallisation of the steroid within the joint, can result in worsening hip pain for up to a week following the injection and can lead to significant morbidity. However, the risks involved in undergoing an intra-articular hip injection are certainly outweighed by those related to operative treatment, especially in cases where it has been found that such treatment would be of no benefit to the patient.

**Fig. 3.**
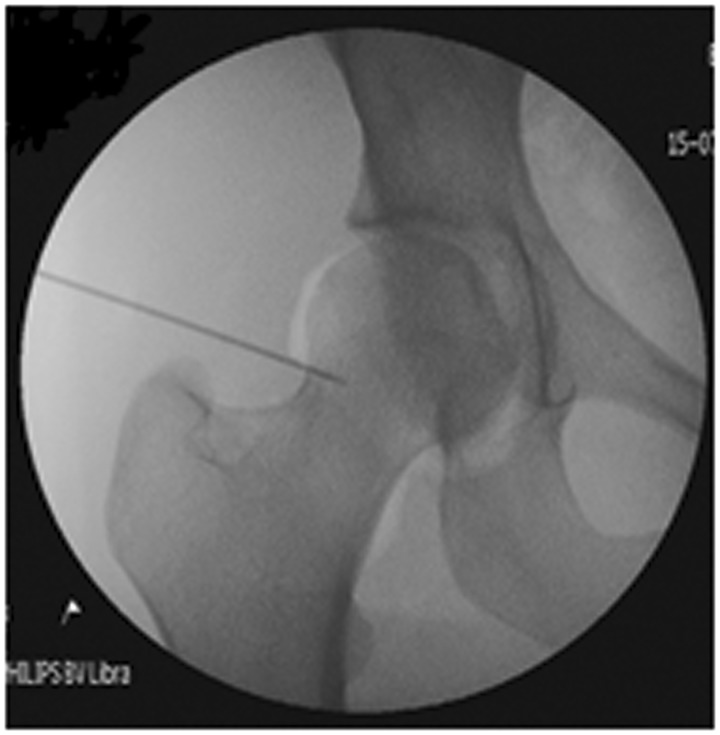
Hip arthrogram.

There are benefits to performing the injections under sedation in theatre. After performing an arthrogram (Fig. 3), the surgeon only infiltrates LA into the joint which does not have an effect on the surrounding structures. Therefore, this is a more sensitive and specific test than an injection done under ultrasound guidance which requires LA infiltration through the surrounding tissues into to the hip joint and relies on the steroid effect which is delayed. Using this technique, the surgeon gathers almost immediate information as to the efficacy of the injection as he can re-examine the patient while the LA has numbed the joint once the patient is awake. Therefore, if the patient's symptoms are extra-articular in origin, the examination will not be temporarily improved. In the case of an ultrasound-guided injection, when assessing the effect the injection has had the surgeon has to rely on the patient being an accurate historian, and a positive response to the steroid injection. Furthermore, theatres are a more sterile environment than clinic rooms, which may reduce the risk of septic arthritis.

In conclusion, hip-type pain in young adults is not always caused by intra-articular pathology even in the presence of abnormalities on examination and/or imaging. An important investigation in the diagnostic pathway is the intra-articular injection. A negative response to intra-articular injection should alert the surgeon to the presence of symptomatic extra-articular causes of hip pain, therefore avoiding unnecessary surgery, its inherent risk and any delays in the treatment of the true pain generator.

## FUNDING

The Elective Orthopaedic Centre, Epsom (EOC).

## CONFLICT OF INTEREST STATEMENT

None declared.
